# Two Faces of Milk Proteins Peptides with Both Allergenic and Multidimensional Health Beneficial Impact—Integrated In Vitro/In Silico Approach

**DOI:** 10.3390/foods10010163

**Published:** 2021-01-14

**Authors:** Anna Maria Ogrodowczyk, Ivan Dimitrov, Barbara Wróblewska

**Affiliations:** 1 Institute of Animal Reproduction and Food Research of Polish Academy of Sciences, Department of Immunology and Food Microbiology, Tuwima 10, 10-748 Olsztyn, Poland; a.ogrodowczyk@pan.olsztyn.pl; 2 Faculty of Pharmacy, Medical University of Sofia, 1000 Sofia, Bulgaria

**Keywords:** allergy, cow’s milk allergens, *in silico* tools, MHC II binded peptides, IL-4 inducing peptides, IFNγ inducing peptides, biologically-active peptides

## Abstract

The main food-origin antigens that the infant’s body is in contact with are cow’s milk proteins (CMP). Still, CMP are one of the main sources of beneficial biologically active peptides that play a role in treatment of non-communicable diseases. Safe methods to quickly predict the sensitizing potential of food proteins among their range of health-promoting properties are essential. The aim of study was to adapt an integrated approach combining several *in silico* (*IS*) studies and *in vitro* (*IV*) assays to screen the multifunctionality of CMP-derived peptides. Major histocompatability complex type II MHC II-binders, interleukin-4 and -10 inducers, interferon γ -inducers and immunobioactivity tools were used to predict the peptide-power of inducing allergies or tolerance. A comparison of the peptide profiless revealed the presence of one identical and one overlapping sequence in *IS* and *IV* hydrolysate. By *IS* analysis, four of 24 peptides were found to have high affinity and stimulate IL-4 expression, and by *IV,* one of seven peptides had this potential (Bos d9 peptide DIPNPIGSENSEK (195–208)). Three *IV* peptides may induce IL-10 expression. The *IV*/*IS* assessment seems promising agents for peptides’ potential determination dedicated only to preliminary screening of peptides. The *IV* verification is still crucial in further steps of studies.

## 1. Introduction

In the 21st century, the world is facing two major problems. On the one hand, the growing population of the world needs a new source of nutritious food [1]. On the other hand, the problem of diet-related diseases and people requiring special medical-purpose food makes it difficult to adapt and use new food sources [2]. The potentially health-promoting activity of food derived peptides seems also to be a top notch aim of studies in that context [3]. However, it is already known that the human immune system is immensely complex but is not always able to develop tolerance to food proteins subjected to various processing technologies [4]. Not entirely known is why developing an immune system that may recognize and respond to infections creates the potential for hypersensitivity reactions. These manifest as allergic responses to environmental agents or autoimmune responses to self-antigens. Therefore, there is a need for an efficient and at the same time safe system to verify the immunoreactive potential of proteins and their resulting peptides [5]. The main food-origin antigens that the infant’s body is in contact with are cow’s milk proteins (CMP). CMP allergy ranges from 0.5% to 3% in developed countries [6]. It has been observed lately that there is an earlier and more severe failure of oral tolerance induction protocols during the first year of life, as well as a likelihood of decrease in the outgrowing of allergy, which forces changes in the approach to conducting therapy [7]. Therefore, highly efficient and at the same time precise and safe methods of testing both new sources of functional proteins/peptides and their usefulness in possible specific immunotherapy are needed.

Along with this, a lot of effort is put into implementing the principles of the 3Rs (replacement, reduction and refinement strategy) in in vivo studies. The effectiveness and repeatability of animal models used to test the allergenic potential of new foods is questionable [8]. Therefore, *in silico* (*IS*) research aimed at faithfully replacing research in this field with the use of animal and cell models can be helpful in the screening of peptides obtained after protein digestion. Each of the available methods has its advantages and disadvantages, and to a varying degree copes with challenges such as cross-reactivity and *de novo* sensitization [9]. Still, the complexity of the immune reactions limits definitive data interpretation so a single analysis may be insufficient. The matter is even more complicated due to several possible mechanisms of hypersensitivity by which the allergy may proceed depending on the dose of the allergen, its digestive resistance, or the route of administration to the body [5,10].

Nevertheless, an allergy is still the adverse response of the body’s immune system to otherwise harmless substances, mostly proteins. The allergenic proteins follow the same path as other foreign antigens that enter the human body. They are broken down into small fragments by human proteases. Some of these fragments bind to human MHC (Major histocompatability complex) class II proteins. A very useful technique to monitor this phenomenon is proteochemometrics [11]. The complexes between the peptides of allergenic origin and MHC class II proteins are expressed on the human cell surface. It is also important to know the alleles of MHC class II involved in the reaction with a specific type of allergen, because the response to individual allergens can be associated with different alleles [12]. The peptide-MHC class II complex can be recognized by antigen-specific armed helper T cells, stimulating them to make proteins that, in turn, cause the B cell to proliferate and its progeny to differentiate into antibody-secreting cells. It is important to understand that even bound epitopes do not always induce the same type of cytokine response. In the context of humoral reaction it is important to verify the potential to induce interleukin-4 (IL4) inducing MHC II binders [13]. The ability and strength of induction of interferon γ (IFNγ) secretion is important in the context of accompanying pro-inflammatory reaction but also, depending on the dose of allergen, its significant role in suppression of the immune system and induction of tolerance to allergenic proteins [14]. Also, interleukin-10 (IL10) induction plays a significant role in tolerance development through the suppressing of inflammatory process, changing the profile of activated effector cells, increasing the expression of proteins forming tight junctions in mucosa and an increased amount of Goblet cells. Reducing the secretion of pro-inflammatory cytokines, e.g., IL-4 and IFNγ with a simultaneous increasing the level of regulatory cytokines (including IL-10) in the context of discrimination of potential of peptides gives hope to more targeted and efficient diagnosis and immunotherapy.

In this article, the authors therefore attempted to develop the algorithm to assess the possibility of an allergic reaction /tolerance to well-known CMP allergens using several *IS* tools effective in vaccine development. Several models in a sequence as a potential pathway to which peptides are subjected in a living organism were begun from simulated digestion. The immunoreactive potential of obtained peptides were determined based on potential to bind MHC class II and IL-4/-10 and IFNγ secretion induction. At the same time beneficial activities of the peptides on the immune system were determined. An analogous procedure was also applied to the cocktail of peptides obtained during the spectral analysis of a cow’s milk sample submitted to simulated digestion by the *in vitro* (*IV*) method.

The authors agree that no existing *IS* solution can replace the *IV* assessment, and cannot be even compared with in vivo approach, but they made an attempt to prepare the theoretical decisive procedure that maximally mimicked conditions prevailing in a living organism. It was decided not to replace *IV* testing but to limit the amount of optional peptides for that procedure. This protocol was especially developed to reduce the use of allergic human sera that are routinely used to test products immunoreactivity. Human sera are invaluable material, and their purchasing is more and more difficult due to the increasing ethical rigors. Also, the sera lifetime and reproducibility are limited so it is necessary to limit to minimum the number of tested formulas.

The main aim was to assess the usefulness of sequential action on *IS* tools in order to find key data that allow determination of whether a specific peptide has a higher sensitizing or modulating potential on the immune system. Such a personalized approach in terms of MHC II alleles can be helpful in predicting the effectiveness and susceptibility to specific immunotherapy and a peptide-based formulas intended for therapy.

## 2. Materials and Methods

### 2.1. Selection of Proteins for In Silico Analysis

**Figure 1:**
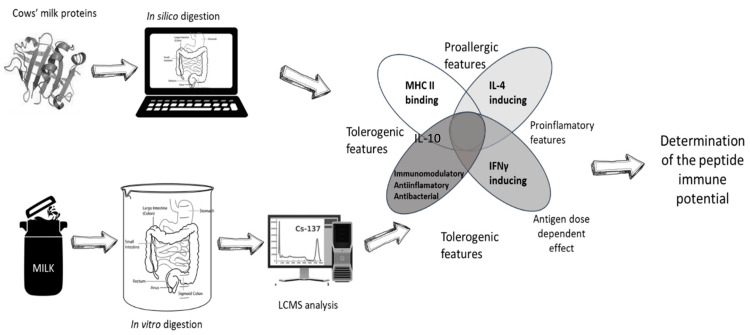
Scheme of *in silico* (*IS*) and integrated *in vitro*/*in silico* (*IV/IS*) analyses.

Major milk allergens were selected based on AllergenOnline version 20 [15] and from Allergome database [16] and the further steps of analysis are shown in Figure 1. Sequences of those allergens were downloaded from Uniprot database [17]. The chosen CMP with allergenic potential and known primary structure (α -lactalbumin (Bos d 4, uniprot id: P00711), β-lactoglobulin (Bos d 5, uniprot id: P02754), serum albumin (Bos d 6, uniprot id: P02769), α -S1-casein (Bos d 9 uniprot id: P02662), α-S2-casein (Bos d 10, uniprot id:P02663), β-casein (Bos d 11, uniprot id: P02666), κ-casein (Bos d 12, uniprot id: P02668) were submitted to further analysis.

### 2.2. In Silico Digestion

*IS* digestion was carried out using PeptideCutter (http://web.expasy.org/peptide_cutter/) as it was described before [12]. Briefly, the protocol mimicking gastrointestinal enzyme digestion with pepsin at pH -2, trypsin and chymotrypsin was carried out to determine a larger pool of possible peptides. The FASTA format of CMP were uploaded on the PeptideCutter server. PeptideCutter predicts potential cleavage sites cleaved by proteases or chemicals in a given protein sequence and returns the query sequence with the possible cleavage sites. After this protocol, each protein is presented as a set of peptides with different lengths. As the peptide-binding groove of MHC class II proteins is able to accept peptides of 9 amino acid residues or longer, in the next step only those peptides were selected and used which resulted from previously established protocols [18].

### 2.3. In Vitro Milk Digestion

Physicochemical properties of commercially purchased 2% fat milk were tested using MilkoScan™ FT2 infrared milk analyzer (Foss, Hilleroed, Denmark). *In vitro* (*IV*) digestion was carried out using Minekus et al., 2014 static *IV* digestion protocol. A simulated gastric and intestinal fluid was added to aliquots of bovine normalized (2% fat, 3.5% proteins, 3.2% lactose) milk [19]. Briefly simulated gastric fluid was added to achieve a concentration of 2000 U/mL pepsin (P700, Sigma-Aldrich, Warsaw, Poland) in the final mixture. After 2h incubation at 37 °C with constant stirring, a pancreatin (P7545, Sigma-Aldrich, Warsaw, Poland) containing simulated intestinal fluid to a final 100 U/mL trypsin activity and bile (B8631, Sigma-Aldrich, Warsaw, Poland) 10 mM concentration was added. After another 2 h incubation under the conditions as specified above the samples were immediately frozen to stop the reaction and freeze dried. The obtained digested samples were used for the subsequent analysis. Undigested milk samples were also lyophilized and subjected to further spectral analysis in an analogous manner. In *IS* models there is unfortunately no option to model digestion of proteins in matrix so in their emulsified form. We need to know then the protein composition that we can separately test in the mathematical *IS* model. That is why we parallel performed an analysis of raw milk (raw material) that was not submitted to *IV* digestion prior MS screening (that allows also to identify trace and labile proteins). Using such a protocol, we get the maximally precise screening of proteins profile in raw material and for further *IS* steps we can choose only those that perform the functions desired for us (like a strong sensitizing potential, or expected tolerogenic potential).

### 2.4. Spectral Analyses of Milk Peptides

Freeze dried samples of proteins and peptides after *IV* digestion were dissolved in 100 μL of 100 mM ammonium bicarbonate buffer, reduced in 100 mM S-Methyl methanethiosulfonate for 30 min at 57 °C, alkylated in 100 mM Tris(2-carboxyethyl)phosphine hydrochloride for 40 min, and digested overnight with 10 ng/mL trypsin (Promega, Madison, WI, USA) at 37 °C. Finally, trifluroaceti acid was added at a final concentration of 0.1%. Mass spectrometry (MS) analysis was performed by online liquid chromatography-mass spectrometry LC-MS in the Laboratory of Mass Spectrometry (IBB PAS, Warsaw, Poland) using a nanoAcquity ultra-performance LC (UPLC) system (Waters, Milford Sound, MA, USA) coupled to an LTQ-Orbitrap Velos mass spectrometer (Thermo Scientific, Waltham, MA, USA). Peptides were separated by a 180-min linear gradient of 95% solution A (0.1% (*v/v*) formic acid in water) to 35% solution B (acetonitrile with 0.1% formic acid). The measurement of each sample was preceded by three washing runs to avoid cross-contamination; the final LC-MS washing run was searched for the presence of cross-contamination between samples. Raw data were searched by Mascot (Matrix Science) against SwissProt database, tax.: Mammals (556 006 sequences). Search parameters: unspecific digest (“enzyme none”) due to simulated digestion pretreatment in hydrolyzed samples and peptides mass tolerance 30 ppm, fragment ion tolerance 0.1 Da, Higher-energy collisional dissociation (HCD) with a normalized collision energy value of 35%, variable modification: methionine oxidation, methylation. The score threshold cut-off- calculated for this analysis was 32. The signals obtained from analysis of hydrolyzed products were compared with signal of proteins and peptides of non-hydrolyzed milk. Obtained peptides were screened in in terms of length to select those structures that also had a minimum of 9 amino acid residues.

### 2.5. In Silico Analysis of Immunoreactive and Bioactive Peptides

#### 2.5.1. The Evaluation of Binding Affinities to MHC II and T- and B-Cells Inducers

Peptides that survived after *IS* and *IV* digestion were tested for binding to human MHC class II proteins. Each peptide was uploaded on EpiTOP3 server [20] (http://www.ddg-pharmfac.net/EpiTOP3/). EpiTOP is a server for human leukocyte antigen (HLA) class II binding prediction using proteochemometric models. It is a web-based application written in Python and HTML and integrating the MySQL database environment. It uses quantitative structure–activity relationship (QSAR) analysis that is a powerful method due to its high and fast throughput and good hit rate. This tool analyzes the primary structure of proteins and predicts potential binders to the most common alleles among human population (12 HLA-DRB1, 5 HLA -DQ and 7 HLA- DP proteins). Based on previous experience, obtained peptides were tested in the context of their binding affinity to DRB1 *01: 01, DQ7 (DQA1*05: 01/DQB1*03: 01) and DQ8 (DQA1*03: 01/DQB1*03: 02) in the context of allergic reactions and DRB1*03:01, DRB1*14:19 and *14:21 which seem protective against cow’s milk allergy [21]. For this confirmation, another *IS* tool (http://tools.iedb.org/mhcii/result/) was used that employs several methods to predict MHC Class II epitopes, including a consensus approach which combines NN-align, SMM-align and combinatorial library methods [22]. Also, B-cell and T-cell epitopes prediction was made using DiscoTope tool and Algpred, respectively. DiscoTope is a method for predicting discontinuous epitopes from 3D structures of proteins in PDB format (http://tools.iedb.org/discotope/). It incorporates solvent-accessible surface area calculations, as well as contact distances into its prediction of B cell epitope potential along the length of a protein sequence. Moreover, thirteen proteins in the training data set were used and prediction methods achieved highly significant predictive performances [23]. Algpred tool (http://crdd.osdd.net/raghava/algpred/) allows prediction of allergens based on similarity of known epitope with any region of protein. It uses a Support Vector Machine (SVM) based method. Five-fold cross validation technique with four sets of data were used for training and testing of this tool by its authors. A hybrid option of analysis was chosen as recommended in [24].

#### 2.5.2. The Determination of IL4 Inducing MHC II Binders

The IL4pred tool (http://crdd.osdd.net/raghava/il4pred/) was used in the next step as a method that can distinguish if the peptide has potential interleukin-4 (IL4) secretion induction. For the analysis, the set of peptides with proven high strength of MHC II binding was used. IL4pred uses also a SVM based method. Five-fold cross validation technique with four sets of data were used for training and testing of this tool by its authors [13]. This approach is important due to the fact that the secretion of interleukin-4 (IL4) is a characteristic of T-helper 2 response, but MHC II epitope binding does not always induce IL-4.

#### 2.5.3. The Determination of IFNγ Epitopes

An online module for the IFNγ inducing peptide/epitope was used (https://webs.iiitd.edu.in/raghava/ifnepitope/). IFNepitope software is an online prediction server that aims to predict and design the peptides from protein sequences having the capacity to induce IFNγ release from CD4+ T cells. This tool also uses a SVM based method [14]. The processes of training and validation of this model are analogous to those described in the IL4pred tool. In this analysis, all peptides obtained during the simulated digestion were tested.

#### 2.5.4. The Determination of IL10 Inducing Binders

The IL10pred tool (http://crdd.osdd.net/raghava/IL-10pred/) was used in the next step as a method that can distinguish if the peptide has potential interleukin-10 (IL10) secretion induction [25]. For the analysis, the set of peptides with proven high strength of MHC II binding was used. IL10pred uses also SVM based method. The processes of training and validation of this model are analogous to those described in the IL4pred tool.

#### 2.5.5. The Determination of Immune-Bioactive Peptides

For this analysis the FeptideDB tool (http://www4g.biotec.or.th/FeptideDB) was used for determination of bioactive peptides with proven benefit for immune system activities. The digestion module in this tool provides its own algorithm for *IS* enzymatic digestion. Steps downloaded from Uniprot database sequences were used. Peptides cleaved from query protein sequences via Pepsin 2pH/trypsin option were analyzed. Here, the same peptides, previously characterized and meeting the length criterion of peptides not smaller than 9 amino acids, were also used for the analysis.

### 2.6. Statistical Analysis and Data Calculation

The *IV* study along with the spectrometric analysis was performed in triplicate. All the data were gathered from separate aliquots of raw material obtained from different containers but purchased at this same time from this same part of dairy beverage (batch no.000237/2018/_G). The *IV* digestion both with spectrometric analysis were conducted parallel. The homogeneity of the model due to the used qualitative analysis of MS allows to estimate the homogeneity based on the presence of peptides in all replicates. The analogical peptides (characteristic for particular protein) found in all the repetitions were tested. The comparison was based on those proteins that were identified basing on peptides considered by Mascot as significant using score value. Basing on indirect quantitative parameters for those peptides the homogeneity of digesta was tested with the Manhattan method of distance analysis where the first dimension was the score value for peptide and the second value was the exponentially modified protein abundance index (emPAI). Based on those variables, the minimal distance between obtained parameters was tested. The accuracy and precision of *IS* models were tested by their creators as described in Section 2.7. The result of integrated analysis was presented in the form of a radial chart where the values for individual variables were normalized to one and the potential was assessed on the basis of the area under the chart. The area was framed by the curve connecting the individual points on the graph. The area saw higher values when several features describing specific properties of the peptides got higher (more significant SVM scores) than the area for a single feature that is presented in the form of a single radius. Factors #4 to #9 were rated as stronger pro-allergic and #10 to #16 as tolerogenic. Factors #1 to #3 as discriminants of weak binging to different alleles of HLA might be significant in both types of reactions. IFN epitopes (#11) no matter if it was stated positive or negative induction were normalized also to one but interpreted in the context of pro-inflammatory activity both with IL-4 and as tolerogenic both with IL-10- however in tested group of peptides the second regularity was not noted. An important role in the nature of the IFN mediated response plays the dose of allergenic peptide that still must be tested empirically.

### 2.7. In Silico Models Training and Validation

The accuracy and precision of the *IS* models were tested by their authors. The developed classification model of IL4pred, IL10pred and IFNγ epitope used the hybrid method of amino acid pairs and motif information. The main dataset used in IL4pred training comprised 904 experimentally validated IL4 inducing and 742 noninducing MHC class II binders. IFNγ contained 3705 IFNγ inducing and 6728 non-IFN-γ inducing MHC class II binders. Another dataset used in training was IFNgOnly, containing 4483 IFN-γ inducing epitopes and 2160 epitopes that induce cytokines other than IFN-γ.

The main dataset used in IL10pred training comprised 394 IL-10 inducing and 848 non-inducing peptides. That approach gave the maximum accuracy of method 75.76% with Matthew’s correlation coefficience (MCC) of 0.51 and 82.10% with MCC of 0.62 for the IL4pred and IFNγ epitopes, respectively. Default settings were used in those systems with SVM threshold: 0.2 and overlapping peptides/epitopes window length: 15 or 9. For Algpred tool, the training and testing process dataset consisted of 578 allergens and 700 non-allergens. The recommended hybrid method enables sensitivity of 93.94% with 33.34% specificity characteristic for motif-based method and 17.47% sensitivity at specificity of 98.14% based on known IgE epitopes [24]. This hybrid method achieves a golden balance between the sensitivity and specificity of individual methods. For the Immune Epitope Database (IEDB) MHC II tool, the recommended 2.22 Consensus approach, combining NN-align, SMM-align, CombLib, and Sturniolo was used [26]. EpiTOP3 returns the binding affinity in pIC50 (negative logarithm of IC50) units. Peptides with pIC50 > 6.3 (IC50 < 500 nM) were considered as strong binders. Peptides with 6.3 >pIC50 > 5.3 (IC50 < 500 nM) were considered as weak binders. In IEDB MHC II tool the predicted output is given in units of IC50 (nM). Therefore, a lower number indicates higher affinity. Peptides with IC50values <50 nM are considered high affinity, <500 nM intermediate affinity and <5000 nM low affinity. The default value of threshold in DiscoTope tool for version 1.1 was −7.7 corresponds to a specificity of 75% and sensitivity of 47% [23].

## 3. Results

### 3.1. In Silico Digestion

Seven bovine milk proteins, allergens with known primary structure underwent IS digestion by pepsin, trypsin and chymotrypsin. Enzyme degradation of allergens significantly reduced the number of peptides with antigenic potential. Digestion of the proteins resulted in 24 peptides longer than nine amino acids (Table 1).

α-lactalbumin (Bos d 4) consists of 142 amino acids. After IS digestion, two peptides longer than nine amino acids have survived: DTQAIVQNNDSTE (56–68) and SSNICNISCDK (88–98).

β-lactoglobulin (Bos d 5) consists of 178 amino acids. After IS digestion, one peptide longer than nine amino acids has survived: ENSAEPEQS (124–132).

Serum albumin (Bos d 6) consists of 607 amino acids. After IS digestion, five peptides longer than nine amino acids have survived: QECCQAEDK (189–197), ICDNQDTISSK (287–297), DAIPENLPPL (319–328), VPQVSTPTL (438–446), CCTKPESER (460–468).

α-S1-casein (Bos d 9) consists of 214 amino acids. After IS digestion, four peptides longer than nine amino acids have survived: DIGSESTEDQAM (58–69), EAESISSSEEIVPNSVEQK (76–94), EIVPNSAEER (125–134), SDIPNPIGSENSEK (195–208).

α-S2-casein (Bos d 10) consists of 222 amino acids. After IS digestion, three peptides longer than nine amino acids have survived: VSSSEESIISQET (22–34), SIGSSSEESAEVATEEVK (68–85), and NAVPITPTL (130–138).

β-casein (Bos d 11) consists of 224 amino acids. After IS digestion, five peptides longer than nine amino acids have survived: NVPGEIVES (22–30), SSSEESITR (32–40), QSEEQQQTEDE (49–59), PFPGPIPNSL (76–85), TQTPVVVPPF (93–102).

κ-casein (Bos d 12) consists of 190 amino acids. After IS digestion, four peptides longer than nine amino acids have survived: GAQEQNQEQPIR (20–31), SCQAQPTTM (108–116), TEIPTINTIASGEPTSTPTTEAVESTVAT (138–166), EDSPEVIESPPEINTVQVTSTAV (168–190).

### 3.2. In Silico Antigenicity Assessment of In Silico Digested Proteins

The 24 peptides originating from IS digestion of milk proteins were tested for binding to the most common HLA-DR, HLA DQ and HLA DP alleles (Table 1).

Bos d 4: The peptide DTQAIVQNNDSTE (56–68) binds to 20 alleles: 7 HLA DP, 5 HLA DQ and 8 HLA DR. It matches a T cell epitope QAIVQNNDSTEYGLFQIN (58–75) and is a part of the known IgE epitope GYGGVSLPEWVCTTFHTSGYDTQAIVQNNDSTEYGLFQINNK (36–77). The peptide SSNICNISCDK (88–98) binds only to HLA DQ alleles. It partially matches a T cell epitope INNKIWCKDDQNPHSSNI (74–91) and is a part of the known IgE epitope GYGGVSLPEWVCTTFHTSGYDTQAIVQNNDSTEYGLFQINNK (36–77).

Bos d 5: ENSAEPEQS (124–132) is a weak binder to 5 DQ and 3 DP allele. Although its sequence couldn’t be matched to any of the known IgE epitopes, it matches a T cell epitope: TDYKKYLLFCMENSAEPEQSL (113–133).

Bos d 6: QECCQAEDK (189–197) shows weak binding affinity only to three of the DQ alleles. ICDNQDTISSK (287–297) binds to 11 of DR, six of DP, and five DR alleles. DAIPENLPPL (319–328) does not bind to any of the studied DR alleles, but shows strong binding affinity to all of the DQ and DP alleles. VPQVSTPTL (438–446) binds to nine DR and all of the DQ and DP alleles. CCTKPESER (460–468) is a strong binder to one DQ allele and a weak binder to one DP and the rest of DQ alleles. None of the peptides that survived after the IS digestion of Bos d 6 are found to match any IgE epitopes. DAIPENLPPL (319–328) is the only binder that matched a T cell epitope: (317–336).

Bos d 9: DIGSESTEDQAM (58–69) binds to five DQ alleles, eight DR and six DP alleles. It is part of the known T cell epitope DIGSESTEDQAMEDIKQMEAESIS (58–81). Parts of it can also be found in two known IgE epitopes, ELSKDIGSES (54–63) and TEDQAMEDIKQMEAE (64–78). EAESISSSEEIVPNSVEQK (76–94) binds to 8 DR, and all DQ and DP alleles. A ten amino acid sequence from it matches the known IgE epitope, EEIVPNSVEQ (84–93), while another part of it matches the T cell epitope PNSVEQKHIQKEDVPSERYLGYLE (88–111). EIVPNSAEER (125–134) binds to five DR, five DQ and seven DP alleles. It matches the known IgE epitope LEIVPNSAEERL (124–135) and the T cell epitope YLGYLEQLLRLKKYKSESTEDQAM (106–129). SDIPNPIGSENSEK (195–208) binds to eight DR, five DQ and seven DP alleles. It matches known IgE epitopes YTDAPSFSDIPNPIGSENSE (188–207), DAPSFSDIPNPIGSENSEKT (190–209) and FSDIPNPIGSENSEKTTMPL (194–213).

Bos d 10: VSSSEESIISQET (22–34) is recognized by all of the DQ alleles, seven DP and three DR alleles. It matches the IgE epitope: KNTMEHVSSSEESIISQETY (16–34). SIGSSSEESAEVATEEVK (68–85) binds to eight DR, five DQ and 7 DP of the studied alleles. Its sequence partially matches two known IgE epitopes: EVVRNANEEEYSIGS (57–71) and EEVKITVDDKHYQKALNEIN (82–101) and the T cell epitope SEESAEVATEEV (73–84). NAVPITPTL (130–138) binds to all DQ and one DP alleles and partially matches a known IgE epitope VPITPTLNREQL (132–143).

Bos d 11: NVPGEIVES (22–30) binds to five DQ alleles and one DP allele. Its sequence fully matches a known IgE epitope, RELEELNVPGEIVESL (16–31). SSSEESITR (32–40) is a weak binder to one DQ and one DP allele. Its sequence matched with a known IgE epitope, LSSSEESITRINKKIEKFQS (31–50). QSEEQQQTEDE (49–59) is a weak binder to all of the studied DQ alleles and one DP allele. Its sequence matched a known IgE epitope, QSEEQQQTEDELQDKIH (49–65). PFPGPIPNSL (76–85) binds to nine DR alleles and all of the DQ and DP alleles. Its sequence matches the known IgE epitope TQSLVYPFPGPIPNSL (70–85). TQTPVVVPPF (93–102) does not bind to DR. It is a strong binder to all of the DQ alleles and a weak binder to all of the DP alleles. Its sequence partially matches a known IgE epitope VVPPFLQPEV (98–107).

Bos d 12 GAQEQNQEQPIR (20–31) and SCQAQPTTM (108–116) do not bind to any of the studied DR alleles, but are recognized by all of the studied DQ and DP alleles. Their sequence does not match any of the known IgE epitopes. TEIPTINTIASGEPTSTPTTEAVESTVAT (138–166) binds to nine of the DR alleles and all of the DQ and DP alleles with high affinity. It partially matches two known IgE epitopes: KKNQDKTEIPTINTIA (132–147) and EAVESTVATLED (158–169).

EDSPEVIESPPEINTVQVTSTAV (168–190) binds to 11 of the DR alleles and all of the DQ and DP alleles. Its sequence almost fully covers the sequence of the known IgE epitope SPEVIESPPEINTVQVTSTA (170–189).

### 3.3. In Vitro Digestion and Peptides Determination

Six allergenic bovine milk proteins, three non-allergenic proteins of cow’s milk and a trace amount of collagen α-1 external contamination in milk were identified after the *IV* digestion by pepsin and pancreatin (Table 2). Pancreatin is a broad-spectrum enzymatic cocktail composed of amylase, trypsin, lipase, ribonuclease and protease obtaining a mixture of peptides even more accurately than with pepsin-trypsin digestion. Enzyme degradation of allergens significantly reduced the number of peptides with antigenic potential and the vast majority of the obtained peptides were shorter than seven amino acids. In in vivo conditions proteins might be less degraded but in static model the digestion of the proteins resulted in seven peptides longer than nine amino acids (Table 2).

α-lactalbumin (Bos d 4)- after IV digestion produced one peptide longer than nine amino acids: NNKIWCKDDQNPSSNICNISCDK (76–98). It contained predicted IS digestion region SSNICNISCDK (88–98).

β-lactoglobulin (Bos d 5) after IV digestion produced one peptide longer than nine amino acids: LVRTPEVDDEALEK-FDKALKALPM (139–163).

Neither serum albumin nor allergen Bos d 6 fragments, longer than nine amino acids, remained after IV digestion. Shorter fragments in spectral analysis did not allow a significant identification of this protein in the sample. The score threshold cut-off-32 calculated for this analysis was still low and proper coverage was not obtained.

α-S1-casein (Bos d 9) after IV digestion produced 4 peptides longer than 9 amino acids. One of them confirmed the IS prognosed sequence SDIPNPIGSENSEK (195–208). The rest were much longer than the prognoses: QKHIQKEDVPSERYLGYLEQLLRL (94–116), GYLEQLLRLKKYKVPQLEIVPNSA (108–131), SMKEGIHAQQKEPMIGVNQELAYF (137–160).

A similar situation was observed for β-casein (Bos d 11). No allergenic fragments longer than 9 amino acids were found but shorter fragments in spectral analysis allowed identification of that protein with 33% coverage. The PAI value indicates that the content of this protein was also consistent with the values accepted for standardized milk.

Neither κ-casein nor allergen Bos d 12 fragments, longer than 9 amino acids, remained after IV digestion. Shorter fragments in spectral analysis did not allow identification of this protein in the sample.

However, the presence of four other CMP characteristic for the milk fat globule membrane (MFGM) *a.o.* was observed (butyrophilin, glycosylation-dependent cell adhesion molecule, osteopontin and allergenic α-1-glycoprotein (Bos d2)). No allergenic fragments longer than 9 amino acids were found but shorter fragments in spectral analysis allowed identifications of those proteins with 5–33% coverage.

During the analysis, the presence of fragments characteristic for collagen α-1was also found. What is more, this protein recognized as allergenic was digested in such a way that one peptide longer than nine amino acids was identified (GPVGNPGPAGPAGPRGEVGLPGLS (266–289)). The PAI value was 0.05 so it was a trace amount, but IS analysis of fragments capable of binding to T and B cells have many inducible regions.

### 3.4. In Silico Antigenicity Assessment of In Vitro Digested Proteins

The 7 peptides originating from IV digestion of milk proteins were tested for binding epitopes for T and B-cells (Table 2) as well to the major groups of HLA-DR, HLA DQ and HLA DP alleles (Table 3).

Bos d 4 peptide NNKIWCKDDQNPHSSNICNISCDK (75–98) binds to 20 alleles: seven HLA DP, five HLA DQ, and eight HLA DR. Although SSNICNISCDK (88–98) peptide binds only to HLA DQ a longer structure binds to seven of the DR alleles, four of the DQ, and three of the DP alleles with high affinity. In Bos d 4 conformational structure, it has 18 regions of potential recognition to T-cells and six to B-cells and in this peptide, so it is six and two, respectively. The peptide SSNICNISCDK (88–98) as was the only one consistent with those predicted in IS digestion and binds only to HLA DQ alleles. In its structure it has only one region of potential recognition to T-cells, but two for B-cells.

Bos d 5 LVRTPEVDDEALEKFDKALKALPM (139–163) is a strong binder to 5 DQ and 7 DP allele but also to 9 DR allele. In Bos d 5 conformational structure, it has 21 regions of potential recognition to T-cells and seven to B-cells, but only four and tow in this peptide, respectively.

Bos d 9 peptide GYLEQLLRLKKYKVPQLEIVPNSA(108–131) is a strong binder to all major tested alleles. In Bos d 9 conformational structure it has 25 regions of potential recognition to T-cells and 12 to B-cells and in this peptide, it is five and two, respectively. SMKEGIHAQQKEPMIGVNQELAYF (137–160) is a strong binder to 5 DQ and 7 DP alleles but also to 8 DR alleles. In this peptide there are only threeregions of potential recognition to T-cells and none for B-cells. QKHIQKEDVPSERYLGYLEQLLRL (94–116) is a strong binder to five DQ and seven DP alleles but also to eight DR alleles. It has six regions of potential recognition to T-cells and three to B-cells.

Collagen α-1 peptide GPVGNPGPAGPAGPRGEVGLPGLS (266–289) is a strong binder mainly to DQ but rather weak for DR alleles. In collagen α-1 conformational structure it has 109 regions of potential recognition to T-cells but only three to B-cells and in this peptide, it is 8 and 1 respectively.

No strong binders for DRB1 * 03:01, and the two similar in structure HLA-DRB1*14:19 and *14:21 and also DRB1 * 04:04, DRB1 * 12:01 and DRB1 * 15:01 have been stated for IS digested peptides but binding of GYLEQLLRLKKYKVPQLEIVPNSA from IV digested peptides with DRB1 * 04:04, and DRB1 * 12:01 was strong (pIC50 −6.658 and 6.315, respectively).

### 3.5. Comparative In Silico Affinity Of Peptides to Chosen MHC II

Affinity of tested peptides to chosen DRB1 *01: 01, DRB1*03:01, DRB1*14:19, DRB1 *14:21 and DQ7, DQ8 is presented in Table 4.

It was observed that only four peptides showed strong affinity to DQ8 belonging to α-S1-casein (Bos d 9) and α-S2-casein (Bos d 10) after IS digestion. Also, both peptides of α-S2-casein (Bos d 10) had strong affinity to DQ7, whereas most of the peptides after IV digestion, except Bos d 9 SDIPNPIGSENSEK peptide, had low affinity (500nM<IC50values <5000 nM). Importantly, calculated affinity for bolded SSNICNISCDK was 30-times stronger than for the longer peptide NNKIWCKDDQNPH**SSNICNISCDK** obtained after IV digestion.

### 3.6. Immunoreactive Activities Of Peptides

All of the described above peptides were submitted to further *IS* analyses to confirm their potential to induce IL-4 and/or IFNγ secretion and to check if there are, in their structures, fragments that can be involved in immunomodulation, antibacterial or anti-inflammatory activities (Table 5).

DTQAIVQNNDSTE peptide belonging to Bos d 4, that has strong affinity to several HLA alleles and T cell epitopes, seems to be unable to induce IL-4 but able to induce IFNγ secretion. In its structure there are no other fragments involved in differential inhibition of Angiotensin-converting-enzyme (ACE) and Dipeptidyl Peptidase IV (DPP IV). On the other hand, SSNICNISCDK peptide is a strong binder only to HLA DQ, but it is a strong IL-4 secretion inducer.

ENSAEPEQS peptide belonging to Bos d 5 that is not able to bind with DRB1 and it is a weak binder to five DQ and three DP alleles, but it seems to have a strong IL-4 expression inductive potential. No other beneficial biological activities of this peptide have been reported so far.

ICDNQDTISSK peptide belonging to Bos d 6 with strong affinity to different types of HLA reveals also a strong potential to induce IL-4 expression but also is able to stimulate IFNγ expression. Only its inhibitory activity to Dipeptidyl Peptidase IV has been reported and deposed in database so far.

DIGSESTEDQAM peptide belonging to Bos d 9, strongly recognized by many HLA alleles, is able to induce IL-4 expression to a moderate degree. In its structure there are previously reported and deposed in database peptides with inhibitory potential to ACE and DPP IV but also stimulating vasoactive substance release. Another Bos d 9 peptide, EAESISSSEEIVPNSVEQK, shows a strong stimulation to IL-4 expression properties and at the same time stimulating IFNγ expression. Moreover, it has been reported to stimulate vasoactive substance release, glucose uptake, as well as thymosin-like peptide which is an immunomodulating activity. In the structure of Bos d 9, both in *IV* and *IS* obtained hydrolysate there was also SDIPNPIGSENSEK peptide. That peptide is a strong inducer of IL-4 expression but also it was reported to have antibacterial activity against *Listeria innocua* and stimulate vasoactive substance release.

VSSSEESIISQET belonging to Bos d 10 has a strong affinity for all tested HLA alleles and it has a moderate potential to induce IL-4 and IFNγ expression. It plays an important role in glucose uptake stimulation. The second tested peptide SIGSSSEESAEVATEEVK that has also strong affinity to tested HLA allele has, at the same time, strong potential to induce IL-4 and IFNγ expression and stimulates vasoactive substance release.

QSEEQQQTEDE peptide, belonging to Bos d 11, is a weak binder and is not reported to be able to induce IL-4 or IFNγ expression. It has no reported activities other than stimulation of vasoactive substance release and DPP IV inhibition.

GAQEQNQEQPIR peptide, belonging to Bos d 12, that in one predictive tool showed no affinity to HLA DR alleles in other models seems to have moderate affinity to some of the alleles. It is also able to stimulate IL-4 expression and its anti-inflammatory and anti-oxidative properties have been reported. Another two peptides from this protein, TEIPTINTIASGEPTSTPTTEAVESTVAT and EDSPEVIESPPEINTVQVTSTAV, show similar anti-oxidative and anti-inflammatory properties but at the same time its fragments EIPT and VQVTSTAV have antibacterial activity against mainly gram-positive bacteria with moderate ability to induce IL-4 and IFNγ expression.

The vast majority of tested peptides obtained during *IV* digestion, except described SDIPNPIGSENSEK, had moderate to weak potential to induce IL-4 and IFNγ expression, but 3 peptides show the ability to induce IL-10 expression.

NNKIWCKDDQNPHSSNICNISCDK peptide of Bos d 4 contains sequences with anti-inflammatory (PHS and TSTA) and antibacterial (CKDDQNPH) activity.

GYLEQLLRLKKYKVPQLEIVPNSA peptide of Bos d 9 contains anti-inflammatory and IL-10 expression boosting sequences (LLR, GYLEQ, RLKKY) and stimulating of glucose uptake dipeptides, or anxiolytic neuropeptides. SMKEGIHAQQKEPMIGVNQELAYF also contains dipeptides with anti-oxidative and anti-inflammatory potential among plenty of DPP IV and ACE inhibitors. One of the Bos d 9 QKHIQKEDVPSERYLGYLEQLLRL peptide contains in its structure the LGY fragment that has proven immunostimulating properties. In that peptide there are also three fragments with anti-inflammatory and one with IL-10 inducing properties.

GPVGNPGPAGPAGPRGEVGLPGLS belonging to collagen α-1, despite its proven allergenic potential, contains many fragments taking significant part in regulation of the stomach mucosal membrane activity, satiety regulation, antithrombotic activity and chemotaxis. With such strong activities, even a trace of such peptides in milk can be significant in inducing reaction.

### 3.7. Integrated Evaluation of Peptides Potential

**Figure 2:**
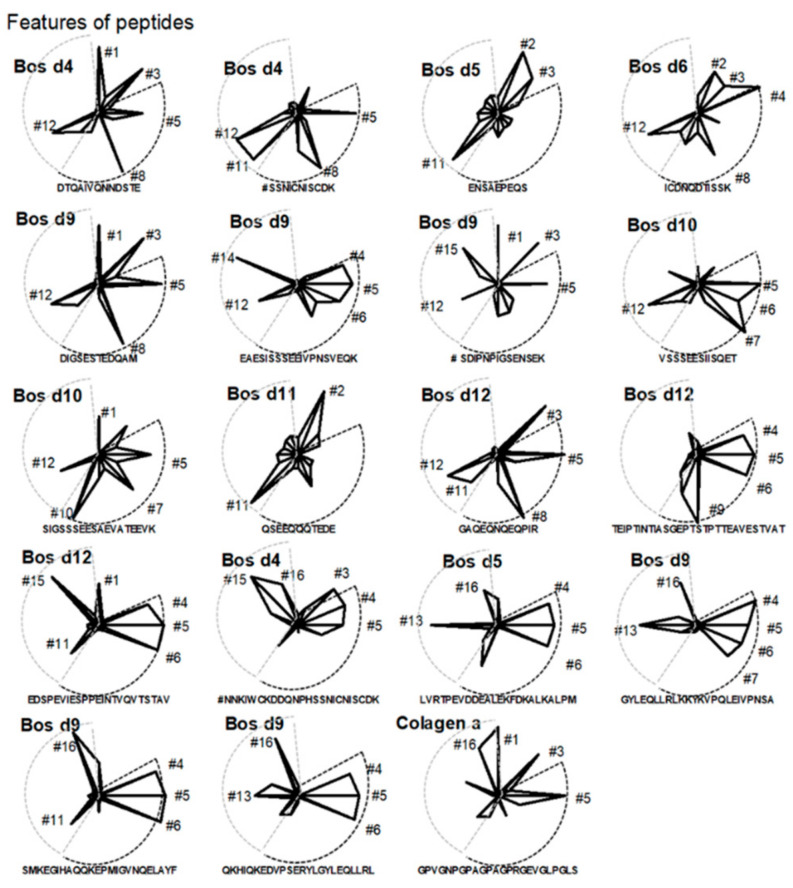
Dominant peptides features integrated with the strength of individual feature in the form of a radial chart.

The assessment was made by comparing the area under the graph prepared for 16 variables described by numerical values obtained thanks to the measurements using the algorithm SVM-score using threshold 0.2 and nine amino acids sequences analysis criterion (Figure 2). Based on this, proceeding peptides like NNKIWCKDDQNPHSSNIC-NISCDK (Bos d4), ENSAEPEQS (Bos d5), EAESISSSEEIVPNSVEQK (Bos d9), and GPVGNPGPAGPAGPRGEVGLPGLS (Collagen α1) can be verified in terms of their moderate tolerogenic potential whereas the rest of peptides needs empirical prove of their allergenicity. Basing on integrated model, peptide SSNICNISCDK (Bos d4), SIGSSSEESAEVATEEVK (Bos d10) and TEIPTINTIASGEPTSTPTTEAVESTVAT (Bos d12) may show particularly strong sensitizing properties. Some peptides like GYLEQLLRLKKYKVPQLEIVPNSA, and QKHIQKEDVPSERYLGYLEQLLRL of Bos d9, despite its potential to induce interleukin 10 expression, show a theoretical strong potential to induce allergies.

The area under the graph is a theoretically determined value based on the SVM score values of individual variables: 1# DRB1-Weak; 2# DQ-Weak; 3# DP-Weak; 4# DRB1-Strong; 5# DQ-Strong; 6# DP-Strong; 7# Strong Allergy involved Allels Inducer; 8# Weak Allergy involved Allels Inducer; 9# IL-4 inducers; 10# IFN gamma inducers; 11# Strong Tolerance involved Allels Inducer; 12# Weak Tolerance involved Allels Inducer; 13# IL-10 inducers; 14# proven Immunomodulative properties; 15# proven Antimicrobial properties; 16# proven Anti-inflammatory properties.

Areas in black dotted line denote strong allergenic features, and areas in gray dotted line, tolerogenic features.

## 4. Discussion

According to the WHO, allergy is currently the third most commonly diagnosed chronic disease [6]. The EAACI report that more than 150 million Europeans suffer from chronic allergic diseases as per the current scenario and half of the entire EU population is foreseen to be affected by 2025 [38]. The consequence of the progression of allergic diseases is a number of possible complications and the well characterized allergic march. However, the latest research mainly focuses on demonstrating possible links between allergic diseases and the development of subsequent eating disorders, psychosocial drawbacks [39] and inflammatory autoimmune diseases [40]. Substantial commonalities between allergy and autoimmune inflammatory diseases in terms of susceptibility loci, genetic pathways, and genomic regulatory sites may also explain the dramatic increase in the incidence of both disorders that are highly heritable. Some commonalities in the genetic architecture of transcriptional cofactors, cell-cycle regulators, regulatory T and helper Th17 cells differentiation factors explain a relationship between these diseases by pinpointing common disease mechanisms that are very complex in both types of diseases [7,39]. Notwithstanding, the only effective way to improve the condition of a patient with a detrimental reaction to food is to eliminate the source of antigen from the diet so far. Such a procedure, regardless of whether it is used during pregnancy, infancy or even adult life, does not always result in a reduction in the frequency of allergic reactions and the development of tolerance. Any deviation and elimination may result in a change in antibody and cytokines profile (including IL-4 and IFNγ) and response to other epitopes [41]. Various dietary changes can induce changes at the epigenetic level. The importance of maintaining allergens consumption, in terms of, e.g., CMP is emphasized for stimulating epigenetic changes that may cause tolerance [7]. As a result of the development of new processing technologies, the structure of some biological food components changes. It is also caused by the attempts to design and develop new multifunctional foods or nutraceuticals.

Animal food proteins are currently the main source of a range of biologically-active peptides which have gained special interest because they may also influence numerous physiological responses in the organism [42,43]. Therefore, it becomes necessary to develop minimally invasive and reliable systems for the initial screening of the properties of the tested peptides [3,5]. Most often, however, large discrepancies appear between *IV* and in vivo tests and even larger ones between tests using *IS* analysis. Numerous predictive models based on various calculation systems and neural networks has been developed so far [9,11,14,18,20,26]. Most often, a single system is used in the analysis. In the present study, an integrated approach combining several *IS* study and *IV/IS* assessment was used to screening the multifunctionality of milk protein-derived peptides. The main focus was on affinity of peptides to MHC II.

The MHC proteins in humans are encoded by the human leukocyte antigen (HLA) system. HLA genes are highly polymorphic which means that they have many different alleles. There are 3 major MHC class II proteins encoded by the HLA: HLA DR, HLA DQ and HLA DP. Not all binders to MHC class II proteins become epitopes but all the epitopes are binders. The structure of the MHC class II proteins’ binding site uses nine amino acids of the peptides’ sequences to bind. Therefore, in order to be recognized by the immune system the peptides should be equal or longer than nine amino acid residues [26,44]. The great majority of *IS* analyses are based solely on assessing the affinity of peptides for individual HLAs but as it was presented also in this study the calculated strength might be different depending on the used predictive method and tool what was observed in our research. Available methods are based on the linear structure of proteins/peptides, or involved more complex computational models, allowing also to take into account the spatial structures of proteins. The support vector machine (SVM) is a tool that allowed integration of properties and determination of regularity that is used in prediction. The difference in this matter to those based in neural network arises depending on the training and testing datasets and ways of developing libraries that reflects on specificity and sensitivity of the model [45]. In this study, almost all of the peptides that originate from *IS* digested allergens bind to all of the five DQ alleles. Taking into account that these alleles are the most common DQ alleles among the human population, man can expect high prevalence of allergic reaction to their parent proteins. The exception, Bos d 11 peptide SSSEESITR (32–40), binds only to one DQ, one DP and one DR allele. The difference in binding affinity to HLA alleles corresponds also to a difference in stability of the complex between allergen and MHC class II protein. Higher affinity supposes more stable complex, hence higher probability a binder becomes a T cell epitope and consequently its parent protein is recognized as an allergen. According to the previous study of the authors it has been stated that there is an association between the susceptibility to milk allergy and the HLA-DRB1/DQ polymorphism. It was stated based on the milk allergen’s strong affinity (average pIC 50 >7, IC 50 <100 nM) to DRB1 * 01:01, DQ7 and DQ8. These alleles could be considered as alleles of susceptibility to cow’s milk allergy. Whereas DRB1 * 03: 01, and two similar in structure HLA-DRB1*14:19 and *14:21 but also DRB1 * 04: 04, DRB1 * 12: 01 and DRB1 * 15:01 have been considered as alleles of protection against allergy due to weak binding to allergens [12,21]. GYLEQLLRLKKYKVPQLEIVPNSA turned out to be an interesting, in this context, peptide that originates from Bos d9. After the *IV* digestion it showed strong binding properties to DRB1 * 04:04 and DRB1 * 12:01. That observation contributed to the search for additional solutions to evaluate other immunological features of tested peptides.

Therefore, it was additionally proposed to verify the allergenic potential of peptides using the analysis of their potential to induce the expression of IL-4 and IFNγ. The secretion of IL-4 is a characteristic of T-helper 2 responses. It has a critical role in guiding antibody class switching, hematopoiesis and inflammation, and the development of appropriate effector T-cell responses. Th2 lymphocytes are formed as a result of the maturation of helper lymphocytes Th0 in the presence of IL-4 produced by previously activated Th2, mast cells, basophils and NKT cells (Natural Killer T cells). IL-4 plays a pivotal role in antibody isotype switching and stimulates the production of IgE [13]. We observed that even peptides defined as strong HLA binders seem not to always show a strong potential for IL-4 expression induction (DTQAIVQNNDSTE) but sometimes seem to induce IFNγ expression. On the other hand, some moderate or weak binders or peptides binding only one HLA allele type like DQ (SSNICNISCDK) show a strong potential to induce IL-4 expression. Thus, IS analysis of the ability to induce IL-4 expression appears to be a helpful tool in assessing the immunoreactive potential of peptides especially in the context of immunotherapy. Also, the role of peptides in IFNγ expression induction seems important due to the fact that IFN plays a significant role in tissue homeostasis, immune and inflammatory responses and tumor immunosurveillance. It is confirmed that it has important implications for autoimmunity, metabolic diseases, atherosclerosis, neurological diseases and immune checkpoint blockade cancer therapy [46]. In that context, the predictive tool both for IFNγ inductors as well as beneficial peptides properties was applied here. Alson IL10pred tool provided some information about tolerogenic potential of peptides. On the one hand, no particularly strong expression-inducing properties were found among the tested peptides but on the other hand plenty of anti-inflammatory dipeptides were present in tested structures.

In the conducted research, it was also important to use a comparative analysis of all features between the profile of peptides obtained during the IS and IV digestion. As has been previously reported by many groups, a small amount of peptides coincide with the obtained profiles. On the one hand, the digestibility of some proteins is very high, so they are quickly digested especially in static model of digestion, into peptides, most of them smaller than 9 amino acids [12,19]. On the other hand, some enzyme cleavage sites are inaccessible, e.g., due to the protective action of other milk components, such as emulsifying lipids [47].

In the mixture obtained after IV digestion one of the peptides belonging to the Bos d 4 allergen (SSNICNISCDK) remained in a longer structure (NNKIWCKDDQNPHSSNICNISCDK). In longer structure both allergic and tolerogenic features changed obtained higher numerical values in the used model for tolerogenic features and lower for allergic. In this context, the use of integrated, sequential IS analysis systems based on IV hydrolysates can be a helpful tool in predicting the potential of peptides and its dynamic changes. However, it should be remembered that it is necessary to test this potential with in vivo methods, but it allows for their preliminary screening. Additionally, the use of sensitive spectrometric methods helps to detect possible contaminants (collagen α-1) that probably got into the product as a result of the technological process of milk standardization. Such a combination of tools and protocols helps in a deeper understanding of various possibilities that cannot be achieved with the use of synthetic peptides. It still seems necessary to validate this approach and continue these studies in term of testing such protocols and their usefulness for other raw materials.

We should also keep in mind that all of these proteins will not be completely digested all of the time in vivo. There are factors such as food components interactions, kinetics involved in the reaction and the intestinal peristalsis constantly moving material through, regardless of whether it is broken down [48]. So, there will be many more peptides longer than 9 amino acid residues presented to MHC-II. The potential differences between the efficiency of digestive enzymes depending on age and health status (like allergy sufferers, people with gastric disorders) [49] will also be an important factor influencing efficiency of proteins degradation. In the context of such predictive difficulties, it would be helpful to include in this type of research a segregated, gastrointestinal flow models of digestion that allow not only to modulate the time of flow and retention of food, but also take into account the volumes of individual organs. Such approaches are improved and increasingly used in drug kinetics research [50]. Thus, they can also contribute to broadening the precision of this approach.

It should also be emphasized that verification of the immunoreactivity of individual model peptides should always be confirmed by IV and/or ex vivo methods, but this publication only aims to develop a method adapted to screening and selection of peptides for those confirming stage of research. Empirical tests of synthetic peptides with cell lines and animal models are expensive, so there is a need to limit choice and variety of tested peptides. Until now, human allergic sera are commonly used, also in our study [51] to verify the immunoreactivity of peptides. Sera remain a unique analytical material, so even the use of extremely sensitive detection methods, such as mass spectrometry, secondary antibodies with fluorescent dyes or quantum dots in immunohistofluorescence [52] does not eliminate the need of immunoreactivity verification with this problematic material which remains well characterized allergic human serum. The proper selection of peptides for further, more sensitive analysis, seems as important as non-targeted IV screening.

Some reports proving the IgE-immunoreactivity of certain peptides (reported in our study) confirmed with the use of human sera, are already available. Still, the etching model used in those publications were limited to single pepsin protocol or trypsin/chymotrypsin assessment. The isolated proteins like α-S1 casein and β- Lactoglobulin were tested, respectively [53,54]. Peptides obtained during that methods differed a bit from ours but mainly in their length and sequence of amino acids but not so much according their immunoreactivity. Some peptides were reported to be IgE-reactive (like: IGVNQELAYFYPELFRQFYQ) [53]. In our model after three-steps of IV digestion we observed the peptide (SMKEGIHAQQKEPMIGVNQELAYF) that contained the fragment IGVNQELAYF. In our study we concluded it might be a strong binder to moderate number of alleles of MHC II but also it was only a moderate inducer of IL-4 related allele. In referred studies of Elsayed et al., 2004 [53] that peptide was inducing only a reaction on moderate level class 1 and 2 of antibody content. That findings seems to be consistent. This same situation was observed with another peptide (DIPNPIGSENSEKTTMPLW) reported by that group as both IgG/IgE reactive and was interpreted as major inhibiting epitope. We also observed that SDIPNPIGSENSEK is a strong inducer of proallergenic and tollerogenic alleles, and a strong IL-4 allele inducer, but at this same time shows reported previously immunomodulative properties. This is a mentioned double face of peptide that can induce both types of reactions.

The results of another group also proved a deep hydrolysis of some allergenic proteins like β-Lactoglobulin and confirmed the lack of some tested, synthesized epitopes like β-Lactoglobulin 102–124. That IV test was consistent with our modelling and confirmed the applicability of IV digestion prior IS assessment utility in predicting and testing possible peptides. Still the low molecular mass of peptides <7kDa with moderate IgE-affinity was reported [54]. All that results show a high level of consistency with our protocol. The predictivity and applicability of our developed theoretical model might be a really promising. The limitation for this model might be all the possible modifications (glycation, phosphorylation and acetylation) that can both reduce or boast the immunoreactivity of peptides and that was already reported [55]. Our study provides a tool dedicated only to preliminary screening. Without a doubt, the further verification of peptides properties must be made, and we do not discuss that here.

## 5. Conclusions

The assessment of the affinity of peptides to MHC class II proteins allows identification of potentially antigenic peptides but still this approach needs to be multi-tool and validated with various methods. We found two common sequences for both IS and *IV/IS* integrated protocols. Both of those structures contain strong binders to MHC class II proteins but only one of them SDIPNPIGSENSEK was 100% identical to *IS* predicted. On the other hand, altering the enzyme cleavage frame may result in a change in the predicted properties of the peptides. The integration of the *in vitro* and IS methods creates a lot of possibilities to faithfully reproduce the reactions taking place in a living organism. To the best of the authors’ knowledge, such a sequential approach has been not applied yet.

## Figures and Tables

**Table 1:** *In silico* antigenicity assessment of *in silico* digested peptides with major MHC II alleles according the EpiTOP3 tool.

Protein	Peptides Surviving after *In Silico* Digestion	Start-End Position	Number of HLA Alleles with Weak Binders *			Number of HLA Alleles with Strong Binders *			T Cell Epitopes ** Start-End Position		IgE Epitopes **	
			DRB1	DQ	DP	DRB1	DQ	DP	Start-End Position	Ref.	Start-End Position	Ref.
Bos d4	DTQAIVQN-NDSTE	56–68	7	1	5	1	4	2	58–75	[27]	36–77	[28]
	SSNICNISCDK	88–98	0	1	0	0	4	0	74–91	[27]	79–98	[29]
Bos d5	ENSAEPEQS	124–132	0	5	3	0	0	0	113–133	[23,24]		[30,31]
Bos d6	QECCQAEDK	189–197	0	3	0	0	0	0	-			
	ICDNQDTI-SK	287–297	2	3	3	9	2	3	-			
	DAIPENLPPL	319–328	0	0	0	0	5	7	317–336	[32]		
	VPQVSTPTL	438–446	6	0	3	3	5	4	-			
	CCTKPESER	460–468	0	4	1	0	1	0	-			
Bos d9	DIGSESTED-QAM	58–69	6	0	5	2	5	1	58–81	[33]	54–63, 64–78	[10,34]
	EAESISSSEE-IVPNSVEQK	76–94	0	0	1	8	5	6	88–111	[33]	84–93	[10]
	EIVPNSAEER	125–134	3	0	6	2	5	1	124–135	[33]	124–135	[10]
	SDIPNPIGSE-NSEK	195–208	8	0	6	0	5	1			188–207, 190–209, 194–213	[10,34]
Bos d10	VSSSEESIISQ-ET	22–34	2	0	2	1	5	5	-		16–34, 28–47	[35,36]
	SIGSSSEESA-EVATEEVK	68–85	5	0	4	3	5	3	73–84	[37]	57–71, 82–101	[34,35]
	NAVPITPTL	130–138	0	1	1	0	4	0	-		132–143	[36]
Bos d11	NVPGEIVES	22–30	0	3	1	0	2	0	-		16–31	[10]
	SSSEESITR	32–40	0	1	1	0	0	0	-		31–50	[34]
	QSEEQQQTE-DE	49–59	0	5	1	0	0	0	-		49–65	[35]
	PFPGPIPNSL	76–85	6	1	0	3	4	7	-		70–85	[10]
	TQTPVVVPPF	93–102	0	0	7	0	5	0	-		98–107	[10]
Bos d12	GAQEQNQE-QPIR	20–31	0	0	5	0	5	2	-			
	SCQAQPTTM	108–116	0	0	7	0	5	0	-			
	TEIPTINTIAS-GEPTSTPTTE-AVESTVAT	138–166	1	0	0	8	5	7	-		132–147	[10]
	EDSPEVIESP-PEINTVQVT-STAV	168–190	4	0	0	7	5	7	-		170–189	[10]

* For alleles with strong binders, weak binders numbers are set to 0; ** Only epitopes that match the peptides are shown. Bos d 4- α-Lactalbumin; Bos d 5- β-Lactoglobulin; Bos d 6- serum albumin; Bos d 9- α -S1-casein; Bos d 10- α-S2-casein; Bos d 11- β-casein, Bos d 12- κ-casein.

**Table 2:** Characteristics of the milk chydrolysate obtained after IV digestion and screening of peptides obtained from MASCOT in terms of inducers for T and B lymphocytes.

Protein	Accession a	Score b	Coverage c [%]	Mass d	PAI e	T Cell Inductor f	Identified Peptides and Its Alergenic Potential/IgE Allergen g	B Cell Inductor h
Bos d 9	P02662	32,934	43	24,570	195.07	25	GYLEQLLRLKKYKV-PQLEIVPNSA;	12
							SMKEGIHAQQKEP-MIGVNQELAYF;	
							QKHIQKEDVPSERY-LGYLEQLLRL; #SDIPNPIGSENSEK	
Bos d 5	P02754	18,489	33	20,269	186.95	21	LVRTPEVDDEALEK-FDKALKALPM	7
	
Bos d 11	P02666	3930	33	25,148	4.25	25	-	13
Bos d 4	P00711	1387	26	16,236	4.99	18	# NNKIWCKDDQNP-SSNICNISCDK	6
Bos d 10	P02663	688	19	26,173	1.61	26	-	12
Osteopontin	P31096	393	33	31,000	1.95	31	-	8
Butyrophilin subfamily 1	P18892	171	5	59,923	0.15	57	-	22
Glycosylation-dependent cell adhesion molecule	P80195	81	23	17,198	1.05	21	-	6
α-1-glycoprotein Bos d2	Q5GN72	74	5	23,158	0.11	40	-	15
Collagen α-1 (III) chain	P04258	71	1	93,708	0.05	109	GPVGNPGPAGPAG-PRGEVGLPGLS	3

^a^ NCBI database accession number; ^b^ Score- The protein score derived from the ions scores of MS/MS report based on the calculated probability, when the significance threshold was chosen to be 0.05, score cut-off-32; ^c^ Coverage- expressed in % number of amino acids in a specific protein sequence that were found in significant peptide matches; ^d^ Mass—Molecular Weight calculated from the database used in this study (http://www.uniprot.org/). ^e^ PAI- Protein Abundance Index in tested sample/Shevchenko et al. (1996). ^f^ T Cell Epitope Prediction binding sites calculated for structure of peptide using MHC II tool http://tools.iedb.org/mhcii/result/. ^g^ Peptides identified in MS analysis and potential allergenicity/IgE allergen- potential predicted allergen based on similarity of identified peptides to the known epitope/protein is assigned allergen if it has a region/peptide identical to known IgE epitopes- based on Algpred tool (http://crdd.osdd.net/raghava/algpred/). ^h^ B cell inductor- binding sites calculated basing on structure of peptide using DiscoTope tool (http://tools.iedb.org/discotope/); # sequence present in the peptide pool after IS and IV digestion. No allergenic fragments of α-S2-casein (Bos d 10), longer than 9 amino acids, remained after IV digestion. Shorter fragments in spectral analysis allowed identification, with 19% coverage, the presence of this protein. The Protein Abundance Index (PAI)-1.61 unit indicates an value equal to the reference mean value of this protein for cow’s milk. (-) no peptide defined as an allergenic.

**Table 3:** IS antigenicity assessment of IV digested peptides with major MHC II alleles according the EpiTOP3 tool.

Protein	Peptides Surviving After *In Silico* Digestion	Start-End Position	Number of HLA Alleles with Weak Binders *			Number of HLA Alleles with Strong Binders *		
			DRB1	DQ	DP	DRB1	DQ	DP
**Bos d 4**	NNKIWCKDDQNPHS- SNICNISCDK	75–98	1	1	4	7	4	3
**Bos d 5**	LVRTPEVDDEALEK-FDKALKALPM	139–163	3	0	0	9	5	7
**Bos d 9**	GYLEQLLRLKKYKV-PQLEIVPNSA	108–131	0	0	0	12	5	7
	SMKEGIHAQQKEP-M**IGVNQELAYF**	137–160	3	0	0	8	5	7
	SDIPNPIGSENSK	195–208	8	0	6	0	5	1
	QKHIQKEDVPSERYL-GYLEQLLRL	94–116	1	0	0	8	5	7
**Collagen α-1**	GPVGNPGPAGPAGP-RGEVGLPGLS	266–289	6	0	4	2	5	3

Bolded peptide determined both in *in vitro* and *in silico* digestion proces.

**Table 4:** IS tested affinity of obtained both (A) IS and (B) IV peptides to selected, defined MHC II according the IEDB Analysis Resource.

Affinity IC50values [nM] Peptides Surviving after Digestion			Involved in Milk Allergy			Involved in Tolerance		
			DRB1 *01:01	DQ7 (DQA1 *05:01/DQB1 *03:01)	DQ8 (DQA1 *03:01/ DQB1 *03:02)	DRB1 *03:01	DRB1 *14:19	DRB1 *14:21
(A) *IS*	Bos d 4	DTQAIVQNNDSTE	121.63	101.36	76.41	93.56	96.68	134.10
		#**SSNICNISCDK**	502.78	308.52	291.38	365.66	445.64	462.78
	Bos d 6	ICDNQDTISSK	462.78	514.2	354.23	234.25	359.94	468.5
	Bos d 9	DIGSESTEDQAM	266.88	240.19	68.2	169.02	287.63	287.63
		EAESISSSEEIVPNSVEQK	462.53	107.92	**16.96**	174.73	277.52	426.55
		# **SDIPNPIGSENSEK**	96.91	67.84	**48.46**	99.07	105.53	106.6
	Bos d 10	VSSSEESIISQET	124.75	**46.78**	**1.72**	102.92	145.02	146.58
		SIGSSSEESAEVATEEVK	272.29	**47.35**	**0.53**	260.45	293.01	295.97
	Bos d 11	QSEEQQQTEDE	531.34	508.49	165.69	565.62	571.34	571.34
	Bos d 12	GAQEQNQEQPIR	278.74	281.70	204.61	275.77	278.74	287.63
		TEIPTINTIASGEPTSTPTTEAVESTVAT	>5000	4253.87	>5000	>5000	>5000	>5000
		EDSPEVIESPPEINTVQVTSTAV	973.05	858.58	629.62	2146.44	2432.64	2690.21
(B) *IV*	Bos d 4	#NNKIWCKDDQNPH**SSNICNISCDK**	4244.21	3480.25	3862.23	3352.93	3650.02	4116.89
	Bos d 5	LVRTPEVDDEALEKFDKALKALPM	1952.34	3268.04	1273.26	2588.97	1527.92	1315.71
	Bos d 9	GYLEQLLRLKKYKVPQLEIVPNSA	1145.94	3013.39	3310.49	2970.95	1612.8	373.49
		SMKEGIHAQQKEPMIGVNQELAYF	3437.81	3310.49	1230.82	1527.92	2376.76	1527.82
		# **SDIPNPIGSENSEK**	96.91	67.84	**48.46**	99.07	105.53	106.6
		QKHIQKEDVPSERYLGYLEQLLRL	1909.9	3055.83	1867.45	2037.22	2970.95	2291.87
	Collagen α-1	GPVGNPGPAGPAG-PRGEVGLPGLS	2776.07	343.43	2747.45	2833.31	2861.93	2861.93

Peptides with IC50values <50 nM are considered high affinity, <500 nM intermediate affinity and <5000 nM low affinity. **Strong affinity bolded**; # sequence present in the peptide pool after IS and IV digestion.

**Table 5:** Immunoreactive activities of peptides.

Peptides Survived after Digestion			IL-4 Inducers ^a^		IFN γ Inducers ^b^		Bioactive Peptides ^c^
			Inductors	SVM Score	Inductors	SVM Score	Function (Number of Sequences)
(A) *IS*	Bos d 4	DTQAIVQNNDSTE	0	-	2	0.33–0.39	-
		# SSNICNISCDK	4	0.99–1.58	0	-	-
	Bos d 5	ENSAEPEQS	2	1.15–2.04	0	-	-
	Bos d 6	ICDNQDTISSK	3	0.52–1.05	3	0.34–0.39	-
	Bos d 9	DIGSESTEDQAM	2	0.21–0.38	0	-	-
		EAESISSSEEIVPNSVEQK	2	0.25–0.99	1	0.75	Immunomod. (3)
		# SDIPNPIGSENSEK	7	0.56–1.66	2	0.07–0.35	Antimicrobial (1) Immunomod. (1)
	Bos d 10	VSSSEESIISQET	1	0.26	2	0.01–0.39	-
		SIGSSSEESAEVATEEVK	5	1.12–1.92	8	0.05–0.72	-
	Bos d 11	QSEEQQQTEDE	0	-	0	-	-
	Bos d 12	GAQEQNQEQPIR	4	0.47–0.8	0	-	Antiinflam. (1)
		TEIPTINTIASGEPTSTPTTEAVESTVAT	14	0.29–1.14	5	0.11–0.31	Antimicrobial (1)
		EDSPEVIESPPEINTVQVTSTAV	0	-	0	-	Antimicrobial (1)
(B) *IV*	Bos d 4	# NNKIWCKDDQNPHSSNICNISCDK	1	0.27	0	-	Antiinflam. (2) Antimicrobial (1) Immunomod. (1)
	Bos d 5	LVRTPEVDDEALEKFDKALKALPM	0	-	5	0.11–0.31	Antiinflam. (2) IL-10 Inducer (2) ^d^
	Bos d 9	GYLEQLLRLKKYKVPQLEIVPNSA	1	0.89	1	0.04	Antiinflam. (3) Immunomod. (1) IL-10 Inducer (2) ^d^
		SMKEGIHAQQKEPMIGVNQELAYF	2	0.32–0.47	0	-	Antiinflam. (3)
		# SDIPNPIGSENSEK	7	0.56–1.66	0	-	Antimicrobial (1) Immunomod. (1)
		QKHIQKEDVPSERYLGYLEQLLRL	1	0.51	2	0.21–0.63	Antiinflam. (3) Immunomod. (1) IL-10 Inducer (1) ^d^
	Collagen α-1	GPVGNPGPAGPAGPRGEVGLPGLS	1	0.54	2	0.04–0.11	Antiinflam. (2) Immunomod. (1)

^a^ IL4pred tool (http://crdd.osdd.net/raghava/il4pred/); ^b^ IFNepitope tool (https://webs.iiitd.edu.in/raghava/ifnepitope/). ^c^ FeptideDB tool (http://www4g.biotec.or.th/FeptideDB); ^d^ IL10pred tool (http://crdd.osdd.net/raghava/IL-10pred/); Immunomod.-immunomodulatory, Antiinflam.- antiinflamatory, Antimicrobial- antibacterial activities. # sequence present in the peptide pool after IS and IV digestion. (-) no peptides showing properties.
